# Leveraging eDNA to expand the study of hybrid zones

**DOI:** 10.1111/mec.15514

**Published:** 2020-07-07

**Authors:** Kathryn A. Stewart, Scott A. Taylor

**Affiliations:** ^1^ Institute for Biodiversity and Ecosystem Dynamics University of Amsterdam Amsterdam The Netherlands; ^2^ Department Ecology and Evolutionary Biology University of Colorado Boulder Boulder CO USA

**Keywords:** environmental DNA, genetic admixture, hybridization, speciation

## Abstract

Hybrid zones are important windows into ecological and evolutionary processes. Our understanding of the significance and prevalence of hybridization in nature has expanded with the generation and analysis of genome‐spanning data sets. That said, most hybridization research still has restricted temporal and spatial resolution, which limits our ability to draw broad conclusions about evolutionary and conservation related outcomes. Here, we argue that rapidly advancing environmental DNA (eDNA) methodology could be adopted for studies of hybrid zones to increase temporal sampling (contemporary and historical), refine and geographically expand sampling density, and collect data for taxa that are difficult to directly sample. Genomic data in the environment offer the potential for near real‐time biological tracking of hybrid zones, and eDNA provides broad, but as yet untapped, potential to address eco‐evolutionary questions.

## 1 INTRODUCTION

Hybridization occurs when evolutionarily independent taxa (groups that differ in one or more heritable characters) interbreed and produce offspring with admixed genomes. Hybridization has long been considered an important window into the ecological and evolutionary processes that determine species dynamics (Harrison, 1990; Harrison & Larson, 2014). The study of hybrid zones—regions in nature where hybridization occurs—has further provided insights into the nature of species boundaries, the role that hybridization may play in adaptive introgression and speciation, and the influences that climate and environmental disturbance have on the distributions and interactions between species (Harrison, 1990; Stewart, Austin, Zamudio, & Lougheed, 2016; Taylor & Larson, 2019; Taylor, Larson, & Harrison, 2015). As the data used to study hybrid zones have shifted towards higher resolution genome‐spanning sets of loci (Gompert, Mandeville, & Buerkle, 2017), we have expanded our understanding of the importance and prevalence of hybridization in nature (reviewed in Taylor & Larson, 2019). Still, our ability to broaden spatiotemporal sampling of hybrid zones and document hybrid zone movement through time has not advanced as rapidly, which limits our ability to fully comprehend the magnitude and consequences of hybridization in nature, especially in the face of rapid anthropogenic influences altering species contact (e.g., climate change, invasive species).

Hybridization is a widespread phenomenon documented across the tree of life (Mallet, Besansky, & Hahn, 2016) and is probably more common than we currently recognize (Levin, 2006). Yet, as rates of hybridization increase globally because of species introductions, range shifts, and anthropogenic disturbances, the accurate quantification of hybridization, the examination of temporal trends in the extent and location of hybrid zones, and the tracking of changes in species interactions at the level of the genome through time, become increasingly important (Buggs, 2007; Grabenstein & Taylor, 2018; Taylor et al., 2015). Although outcomes of hybridization are variable—both positive and negative from an evolutionary or species conservation perspective (Grabenstein & Taylor, 2018)—without accurate documentation, we cannot determine the consequences of hybridization, or mitigate hybridization in instances where it threatens species survival. Thus, despite renewed calls for temporally repeated and high‐resolution studies of hybrid zones, our ability to thoroughly investigate the dynamics within hybrid zones has been limited by various factors.

Despite its widespread nature (Mallet et al., 2016) and ecosystem altering consequences (Taylor & Larson, 2019), our current understanding of hybridization in nature is often restricted to morphologically distinct, or ecologically disparate, abundant taxa predominantly in temperate regions (McEntee, Burleigh, & Singhal, 2018). Most hybrid zone studies are also conducted in a single season, across a single geographic replicate. Given our growing awareness that hybridization between the same taxa can have variable outcomes that depend on geography, ecology/ life history, local demographics, and habitat, (e.g., Mandeville et al., 2017; Schumer et al., 2017; Stewart, Hudson, & Lougheed, 2017; Stewart, Ma, Zheng, & Zhao, 2017), such studies limit our ability to draw broad conclusions about evolutionary and conservation related outcomes of hybridization. While many would prefer to incorporate repeated geographic and temporal sampling into studies of hybridization, the reality of short funding cycles, logistical challenges of geographically replicated field work, and sequencing costs for thousands of samples, has limited the number of temporal or geographically replicated investigations of hybrid zones (see Buggs, 2007).

Genomic sequencing techniques and their decreasing costs have partially alleviated this problem, even for non‐model organisms, bringing such studies within the realm of possibility for most labs. However, replicated sampling at the scale needed to adequately address questions about the consistency of interspecific interactions in hybrid zones remains challenging, especially for organisms that are logistically difficult to directly sample. A potentially powerful approach is to apply innovative tools to uncover this hidden information, and thus increase the efficiency, accuracy, repeatability, and comprehensive nature of sampling hybrid zones, especially during early gene exchange. Environmental DNA (eDNA), combined with existing genomic resources for hybridizing species, in certain systems, has the potential to expand our understanding of hybridization in nature.

## 2 USING AN INNOVATIVE SAMPLING APPROACH TO STUDY HYBRID ZONES

An exciting new molecular avenue to study hybrid zones could be the collection of environmental DNA, or “eDNA”. eDNA is DNA that resides in, and is subsequently collected and extracted from environmental samples. It affords a means of collecting information without visual observation or direct handling of organisms, the latter of which can have negative impacts on the organisms or the habitats in which they live and requires expertise and spatiotemporal sampling effort (Jerde, Mahon, Chadderton, & Lodge, 2011). Sometimes referring to samples obtained from direct remains (e.g., hair, saliva, scat), much work utilizing eDNA uses indirect genomic remnants found within the environment (e.g., air, water, or soil) which allows for sampling areas of suspected site occupancy and increased access to habitats that are difficult to sample. Whether subcategorized into intracellular (e.g., DNA enclosed within cell membranes) or extracellular (e.g., free‐floating nucleic acids after cell lysis), eDNA represents a biological archive of genes, species, and communities that historically or currently reside within specific habitats. Although challenges remain, a number of studies have successfully (and repeatedly) used eDNA in both aquatic (e.g., Deiner, Fronhofer, Mächler, Walser, & Altermatt, 2016; Kelly, Port, Yamahara, & Crowder, 2014; Ma et al., 2016; Pilliod, Goldberg, Arkle, & Waits, 2014; Stewart, Hudson, et al., 2017; Stewart, Ma, et al., 2017; Thomsen et al., 2012) and terrestrial (e.g., Andersen et al., 2012; Franklin et al., 2019; Ushio et al., 2017) habitats for occurrence (presence/absence) and relative abundance measures (number of sequenced eDNA reads) (reviewed in Barnes & Turner, 2016; Goldberg et al., 2016; Stewart, 2019). Rapid advances in the use of eDNA have also seen noninvasive sampling markers evolve from mtDNA barcodes of various sizes (Egan et al., 2013; Foote et al., 2012; Ma et al., 2016), to diagnostic SNPs (Uchii, Doi, & Minamoto, 2016; Uchii, Doi, Yamanaka, & Minamoto, 2017), and nuclear DNA (nDNA; Aylward, Sullivan, Perry, Johnson, & Louis, 2018; Bylemans et al., 2017; Carpenter et al., 2013; Dysthe, Franklin, McKelvey, Young, & Schwartz, 2018; Minamoto et al., 2017; Sigsgaard et al., 2020), as well as the employment of CRISPR‐Cas technology for species identification (Williams et al., 2019), making the detection of even closely related species, and their potential admixture, possible.

Building from recent advances in the use and study of eDNA that expand beyond mitochondrial barcodes, we believe that the analysis of eDNA is a potentially powerful tool that could augment studies of hybridization and hybrid zones in nature. Studies of hybridization and hybrid zones could use the collection eDNA to increase temporal sampling (contemporary and historical), to refine and geographically expand sample collection for well‐characterized systems, and to collect data for taxa that are otherwise difficult to directly sample (e.g., rare, cryptic, or otherwise elusive). Three recent reviews have highlighted new potential uses of eDNA, encouraging a transition from strictly taxonomic monitoring and conservation management, to more ecological (Bálint et al., 2018) and population oriented avenues of research (Adams et al., 2019; Sigsgaard et al., 2020). We add to this discussion by suggesting that the analysis of eDNA is a promising tool for evolutionary investigations, particularly for studying hybrid zones. Interestingly, to our knowledge, although a limited number of recent studies have used eDNA approaches to examine potential areas of hybridization in nature (see below), no study has yet used eDNA to estimate admixture. Unquestionably, research avenues regarding hybrid zones and admixture have remained restricted in scope in the emerging field of eDNA.

The use of eDNA for the detection of macroorganisms is especially significant in monitoring invasive genotypes (Ficetola, Miaud, Pompanon, & Taberlet, 2008), which is comparable to documenting parental species genotypes in contact zones. Due to the incredible sensitivity and rapid accumulation of eDNA for occupancy patterns, in near real‐time, it should provide an excellent tool for the quantification of low‐density, transient, or cryptic species, factors that have traditionally made studying hybrid zones challenging. Ideal hybrid zone sampling frameworks are often difficult to accomplish because many clades along the speciation continuum are poorly understood, including their ecology, phenology, breeding behaviour, and how these might differ during divergence; here, we argue eDNA sampling may alleviate some of these difficulties.

## 3 EXPANDING THE GEOGRAPHIC EXTENT AND TEMPORAL RESOLUTION OF HYBRID ZONE STUDIES

**FIGURE 1:**
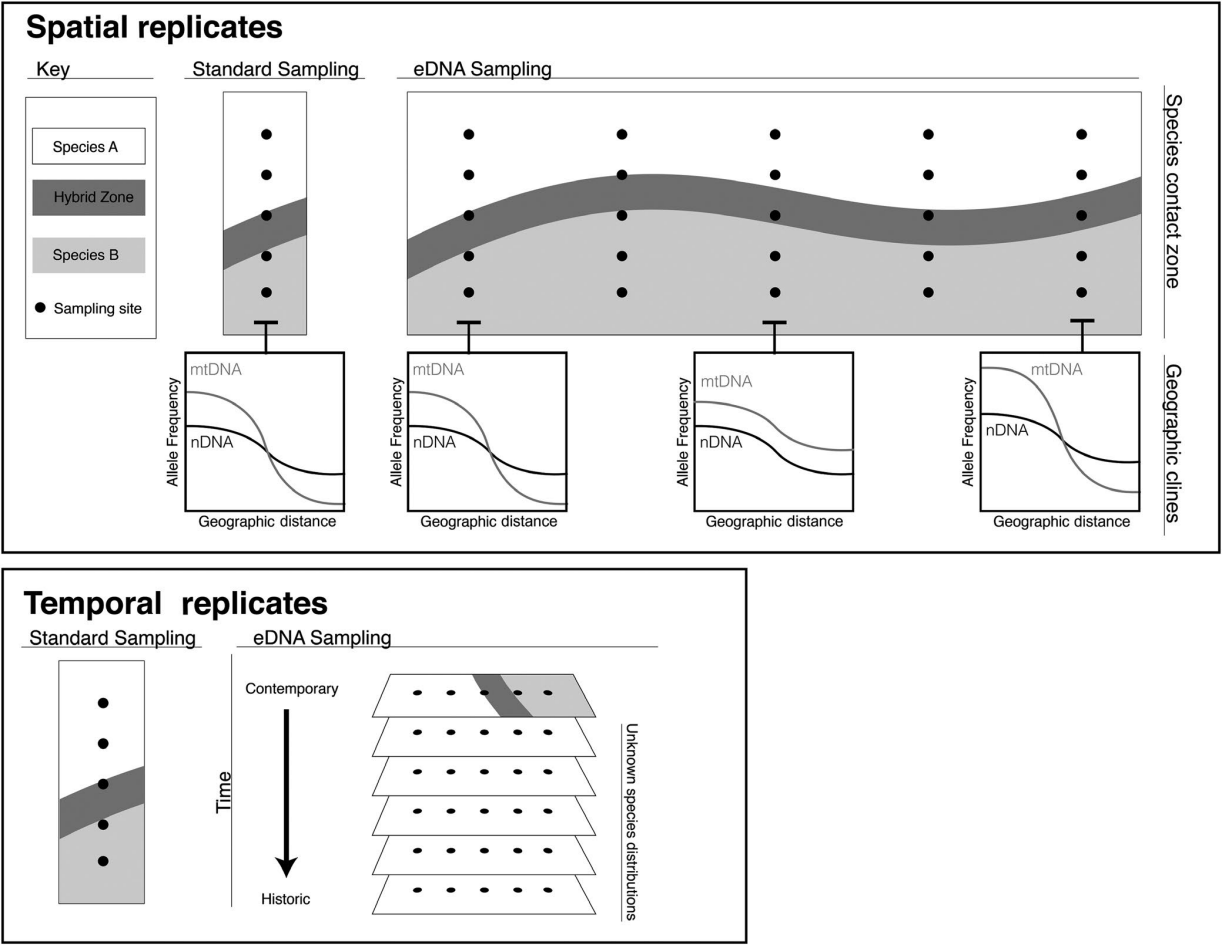
Examples of how spatial and temporal eDNA sampling could facilitate hybrid zone research, including expanded geographic replicates, population‐level cline analysis (mitochondrial DNA, mtDNA; nuclear DNA, nDNA), and comparisons of contemporary and historical samples for the detection of unknown species distributions. Diagram key is located in the top left corner

The majority of hybrid zone studies are geographically restricted and present a single year of data. Given that outcomes of hybridization vary geographically and temporally, this remains a problematic approach. We suggest that one of the biggest contributions eDNA could make to the study of hybrid zones is vastly expanding both the geographic and temporal scopes of hybrid zone studies (Figure 1). This would only be possible for organisms with certain life history characteristics (e.g., standing water aquatic habitats, low dispersal terrestrial organisms, among others).

Collecting DNA from the environment, rather than directly from organisms, can provide high‐resolution temporal data across a large taxonomic breadth and geographic context compared to traditional methods which rely on the direct sampling of organisms (Bálint et al., 2018). At present, most hybridization studies focus on temperate species with obvious morphological or ecological differences (McEntee et al., 2018). With eDNA, previously difficult to study hybridizing taxa, and locations that are difficult to sample for a variety of reasons (e.g., cost, terrain), will provide additional insights into hybridization patterns and processes. For rare individuals or cryptic populations (e.g., juvenile forms), low probabilities of detection increase systematic errors and hinder accurate occurrence estimations, but eDNA sampling efforts increase detection rates, reducing false negatives and confirm true absence records (Wilcox et al., 2018). Further, eDNA collection is both labour, time, and cost‐efficient (Qu & Stewart, 2019), and the collection of eDNA has frequently been included in citizen science projects (e.g., Biggs et al., 2015; Buxton, Groombridge, & Griffiths, 2018), or accomplished via extensive collaborative networks (Wilcox et al., 2018). These aspects alone would vastly improve both the geographical extent and temporal resolution of sampling across hybrid zones, particularly for complex mosaic hybrid zones (e.g., Larson, Andres, Bogdanowicz, & Harrison, 2013) or hybrid zones that extend across national borders (e.g., Ryan et al., 2018; Stewart et al., 2016) and/or have broad geographic distributions (e.g., Scriber, 2011). The ease of collecting environmental samples (e.g., water or soil) further means that dense geographic and repeated temporal sampling could refine known hybrid zone boundaries and identify new regions of contact, while simultaneously allowing for broader sampling coverage without being prohibitively expensive or labour‐intensive.

Moreover, although eDNA molecules often degrade rapidly in nature (on the scales of days to weeks) making eDNA approaches an ideal tool to monitor the contemporary distribution of organisms (Goldberg et al., 2016), eDNA can be successfully amplified up to 1 million years after it is shed into the environment (Kirkpatrick, Walsh, & D'Hondt, 2016; Willerslev et al., 2007). When combined with dating methods (e.g., isotopic analysis, rare historical events that leave paleoecological traces, or annual lamina in sediments; reviewed in Bálint et al., 2018), eDNA may illuminate the historical spatial legacy from species movements. For example, a recent study successfully used eDNA to identify a historical invasion front, contrasting the ecological impact of the invasive species to recent climate change events (Ficetola et al., 2018). Importantly, even the contemporary collection of eDNA can allow for a retroactive look at spatial patterns of occurrence and relative abundance in genes and species through time, which has obvious application to the study of hybrid zones. Aspects of hybridization history and hybrid zone movement, which are often difficult to deduce (e.g., source and speed of admixture, the frequency of reticulated contact, or establishment of tension zones), could all be addressed using spatially and temporally explicit eDNA collections.

Making predictions about hybrid zone movement is also possible when using eDNA tools for hybrid zone investigations. Species distribution models (SDMs) can link biological observations, geospatial habitat, and climactic covariates to forecast future distribution probabilities based on eDNA data (Muha, Rodríguez‐Rey, Rolla, & Tricarico, 2017; Wilcox et al., 2018). By using similar techniques, one could geographically sample hybrid zones, along with the abiotic and biotic parameters that they are correlated with at high‐resolution, and then predict ecologically realistic patterns of introgression and movement trajectories through time. This is an especially useful opportunity for analysing dispersal pathways (Muha et al., 2017) as introgression from introduced species (e.g., Hohenlohe et al., 2013) and climate change (see Taylor et al., 2015) alter species interactions and distributions.

### 3.1 Providing insight into cryptic aspects of hybridization and ecology

We further envision that eDNA can serve as a springboard for the collection of otherwise difficult to sample data. Although our current understanding is that eDNA derives from both dead (e.g., Dell'Anno & Danovaro, 2005; Pietramellara et al., 2009) and living (e.g., Pochon, Zaiko, Fletcher, Laroche, & Wood, 2017) biomass, quantifying viability and fecundity dynamics within hybrid zones might also be tractable with eDNA. Sources of genetic material within environments are varied (Stewart, 2019) and intracellular or eRNA sources are assumed to originate from metabolically‐active living organisms before being rapidly removed from the environment. Examination of proportions of eDNA/eRNA (e.g., Steven, Hesse, Soghigian, Gallegos‐Graves, & Dunbar, 2017), or intra‐ to extracellular eDNA (correcting for degradation), could allow for inferences related to general patterns of mortality either due to hybridization itself (combined with species‐diagnostic sequencing), or via species interaction and competition. If sampling is directed at discrete life‐stages (e.g., egg, larval, and adult forms) that occupy distinct temporal (e.g., seasonal) or geographical (e.g., terrestrial vs. aquatic, or species‐specific aquatic vertical distribution of gametes or eggs; Stewart, 2019) realms, eDNA collections may also open windows into differential mortality throughout development, a central tenet of hybridization research.

The detection of eDNA is also known to spike in aquatic environments during reproductive seasons (e.g., Laramie, Pilliod, & Goldberg, 2015; Spear, Groves, Williams, & Waits, 2015), with breeding events characterized by higher nDNA relative to mtDNA, facilitating quantification of reproductive bouts within hybrid zones and phenological breeding patterns in the parental species coming into contact. As X‐ and Y‐ linked markers (e.g., Brinkman & Hundertmark, 2009; Taberlet, Mattock, Dubois‐Paganon, & Bouvet, 1993), and sex‐associative mtDNA heteroplasmy markers (Mioduchowska, Kaczmarczyk, Zając, Zając, & Sell, 2016) have also been developed for noninvasive sampling, researchers may also be able to determine sex ratios within populations that have genetically determined sex. This is especially important for species that do not display sexual dimorphism. Sex‐linked markers could further provide insight on postzygotic reproductive isolation, such as hybrid dysfunction (Haldane's rule). Likewise, eDNA would allow the quick retrieval of diagnostic genes that differ between the parental species within hybrid zones when accompanied with high‐quality reference genomes and initial exploratory work.

## 4 PRACTICAL CONSIDERATIONS AND POTENTIAL LIMITATIONS

### 4.1 Current challenges and solutions with eDNA

Collections using eDNA molecules are not without their faults, including false‐negative detections even, although rarely, in the presence of focal specimens (e.g., Pinfield et al., 2019). However, false negatives are not restricted to eDNA approaches. False negatives are also a problem with traditional approaches to hybrid zone analysis for some species. For example: (a) behavioural exclusion from breeding, or mortality prior to distinguishable breeding cycles, might prevent individuals from being sampled and thus lead to a misrepresentation of population dynamics within a hybrid zone; (b) the focus on a single life‐stage (usually adult breeders) may also underreport the actual extent of hybridization or fail to document wasted reproductive effort; and (c) biases in capturing methods for adults could also misrepresent hybrid zone dynamics. Finally, cryptic admixed individuals may be morphologically indistinguishable from parental species and not targeted for sampling, but this could be captured, even at very fine scale, using eDNA analyses with the caveats described below.

Transportation and degradation of eDNA molecules are additional concerns. In discrete populations (e.g., lakes, ephemeral ponds), eDNA sampling should include multiple geographic replicates to collect as much information as possible to adequately represent the sampled site, taking into account the ecology of eDNA molecules for that specific taxon (e.g., signal radius, molecular transportation or dispersal). Degradation of eDNA signals also occurs and eDNA samples should be collected within a temporal framework that maximizes contemporary acquisition (e.g., during a breeding season, during migration). Across continuous land(aqua)scapes, population sampling should be conducted on a scale informed by taxon‐specific dispersal ability, or other biologically relevant criteria.

When coupled with proper sampling strategy and marker design, eDNA is robust with low error rates. However, most eDNA studies to date have employed mtDNA as their marker of choice, allowing for the delineation of maternal lineage or contact boundaries, but failing to incorporate aspects of admixture. This is not a problem for determining where overlap between two mitochondrial types (i.e., the location of a potential contact zone) may occur. Indeed, it is a necessary first‐step in studies of hybrid zones to determine where species ranges overlap and where introgression may transpire (Figure 1). The relative proportions of genetically similar taxa can be quantified using mtDNA SNP detection via eDNA sampling (e.g., Uchii et al., 2016, 2017), but key information regarding the dynamics of species interactions, such as hybridization, would remain unavailable. However, eDNA collections quantifying nDNA have now been used successfully in the field (Dysthe et al., 2018; Minamoto et al., 2017), and could reveal important spatiotemporal patterns in areas of contact. By combining different markers (see below for details on potentially useful genetic markers), researchers could perform population level analysis (Figure 1). Because the pool of eDNA data would represent an amalgamation of all individual sequences within a population, analyses could draw from Pool‐Seq pipelines (e.g., Pfenninger et al., 2015; Taus, Futschik, & Schlötterer, 2017), which has been previously suggested (Sigsgaard et al., 2020).

Pool‐Seq has been used to successfully map allele‐frequency changes, as geographic clines, across hybrid zones (e.g., Rafati et al., 2018) for both autosomal and sex‐linked loci. Pool‐Seq approaches are comparable to individually sampled and sequenced approaches, with respect to estimating allele‐frequencies within a sampled population, which is an important component of the study of hybrid zones (Rafati et al., 2018). Importantly, eDNA has been found to be just as accurate for genotyping individuals compared to traditional individual‐based methods of sampling, and can even be used for precise parentage analysis given the right environmental conditions (Holman, Hollenbeck, Ashton, & Johnston, 2019). Having *a priori* knowledge about the frequency of SNP variants, and knowing which SNPs are species or subspecies diagnostic, would be critical for clinal analyses using eDNA. Arguments could be made that compared to traditional sampling, Pool‐Seq may have limited resolution due to the number of aggregate samples (localities) and the degree of allele‐frequency changes across a land(aqua)scape, an issue that could undoubtedly be resolved through extensive but easily attained eDNA collections.

Recent sequencing advances have further demonstrated that accurate estimates of the number of individuals contributing to a sample comprised of DNA from multiple individuals are possible (Sethi, Larson, Turnquist, & Isermann, 2018), which will provide an opportunity for eDNA to inform population ecology. Determining the number of contributing individuals is achieved by examining the relationship between allele counts (based on ploidy level) and the number of genetic contributors within a sample. Statistical models using probabilistic frameworks can then provide likelihood‐based inferences of number of contributing individuals, a method already extensively used in forensic DNA studies (e.g., Curran, Triggs, Buckleton, & Weir, 1999; Haned, Pène, Lobry, Dufour, & Pontier, 2011; Weir et al., 1997) but just entering the field of wildlife ecology (Sethi et al., 2018). Similarly, linkage disequilibrium (LD, a measure of allele association) is an important population genetic statistic that reflects rates of recombination between loci, thus forming the basis for tests of selection, estimates of demography, and signatures of introgression across hybrid zones. Examining LD in Pool‐Seq data has been informative for hybrid zone delineation (e.g., Feder, Petrov, & Bergland, 2012), and similar analyses might help to build a foundation for examining eDNA collections of entire populations to understand demographic processes, such as hybridization, rather than merely the overlap of parental populations (Feder et al., 2012).

### 4.2 eDNA solutions for studying hybrid zones

Importantly, the incorporation of eDNA into studies of hybrid zones will require careful thought and will not be possible for every hybridizing species pair. Of course, genotyping species‐diagnostic mitochondrial markers alone will not suffice, but may be an important first step for determining where proportions of diagnostic mitochondrial markers suggest range overlap between species. In all cases, a reference genome will be required, but the generation of reference genomes for many non‐model organisms is now common practice. Typically (but not always), eDNA is degraded and in short fragments. As such, it might be difficult to assign multiple SNP variants to a single individual. To solve this issue, it would be prudent to sequence a specific region of the nuclear genome previously identified as possessing a high proportion of species‐diagnostic SNPs from whole genome or reduced representation sequence data. Given the genetic architecture that has been described for many hybridizing species (i.e., tight clusters of highly divergent diagnostic regions) this is feasible for many systems.

The rapid development of long read sequencing data (e.g., nanopore technology) also has the potential to make eDNA an attractive choice for the study of hybrid zones because long read data from the nuclear genome would allow researchers to discriminate between haplotypes. Methods of sequencing now standard in population genetics (e.g., reduced representation genome sequencing, shotgun genome sequencing) will not be applicable to eDNA samples because, in most cases, DNA concentrations are too low. However, target capture methods have the potential to solve this problem (Sigsgaard et al., 2020). Target capture would be particularly useful if diagnostic regions of the genome were first identified using high‐quality genomic data from tissue samples, and subsequently used to design probes that target small stretches of highly divergent regions of the genome. Target capture combined with long read sequencing could then allow eDNA to make significant contributions to our understanding of hybridization dynamics in nature. To ground‐truth eDNA methods in hybrid zones, experimental mesocosms could be used for many aquatic and some terrestrial taxa (see Sigsgaard et al., 2020).

Beyond considerations of marker development and use, eDNA may alleviate other challenges associated with studying hybrid zones. Traditional approaches for quantifying hybridization in nature often involve the initial identification of potential hybrid zones using morphology or behaviour to categorise phenotypically intermediate individuals, or phylogeographic and population genetic analyses to identify areas of contact. Subsequently, individuals within these regions are collected and measured across a transect, either opportunistically or within discrete populations, in an attempt to sample as many individuals as possible. These data points are analysed individually and then aggregated at the population level using geographic and/or genomic analyses to create averages within populations (or sliding geographic windows) across diagnostic markers, ultimately front‐loading sampling pipelines (collecting and analysing individuals) before hybrid zones are fully delineated. Thus, for traditional approaches, there further exists both a monetary and computational trade‐off between the number of individuals sampled and the number of loci investigated (Payseur & Rieseberg, 2016). With eDNA collections (e.g., via target capture, Carpenter et al., 2013), areas of suspected hybridization can be sampled quickly and efficiently via population averages directly, and with little sampling effort. In this way, eDNA collections have the potential to reduce the amount of initial work associated with traditional sampling practices. Areas of suspected hybridization during analysis (e.g., regions with discordance between nuclear and mitochondrial markers, increases to LD) can then be more closely inspected for further evidence of hybridization (sampling individuals for morphological and genetic evidence of introgression). We propose that the incorporation of eDNA into studies of hybridization will speed up and expand the geographic breadth of hybrid zone detection. Although individual measures of hybridization (F1, F2, and backcrosses) are, at present, not incorporated into eDNA frameworks, exploratory analyses using eDNA would decrease guesswork in geographic sampling, greatly assisting the ability to pinpoint populations of importance. For refining and expanding sampling for well‐studied hybrid zones with *a priori* information about admixture, eDNA also represents a potentially valuable addition to current sampling protocols. We emphasize that the analysis of eDNA, like many other tools, should not be used as a standalone method for the study of hybridization and hybrid zones in nature. eDNA methodologies have always been used as a compliment to other traditional sampling practices, whether for biodiversity monitoring to confirm presence/absence assays, or in this case, to clarify levels of admixture.

To date, aside from well explored eDNA presence/absence or abundance examples that provide invaluable information, preliminary studies have also successfully used eDNA approaches to deduce population dynamics (Sigsgaard et al., 2017) and hybridization processes between subspecies (Dufresnes et al., 2019; Gorički et al., 2017; Uchii et al., 2016). One recent example of eDNAs utility for hybridization research includes a comparative study validating eDNA metabarcoding technology to unravel cryptic invasions and possible hybridization; suspected zones of hybridization were subsequently confirmed via individual based multilocus population genetic protocols (Dufresnes et al., 2019). eDNA tools have also been used to determine colonization history of non‐native species, native species diversity, and the biotic interactions therein (Nelson‐Chorney et al., 2019). This use of eDNA methods ultimately emphasized biogeographical processes, further highlighting how eDNA tools can shed light on previously intractable questions about historical distributions that, until now, have remained primarily qualitative (Nelson‐Chorney et al., 2019).

### 4.3 Environmental DNA analyses are currently underutilized tools for studying hybrid zones

Genomic data from the environment offer the potential for near real‐time biological tracking. Since its inception for macro‐organismal use (Ficetola et al., 2008), eDNA analysis has been widely adopted and utilized in conservation biology, although it provides broader yet untapped potential to address eco‐evolutionary questions. eDNA tools are especially useful for detecting cryptic species and unique genotypes. Thus, a promising application for eDNA in an eco‐evolutionary framework is to obtain quantitative measures of species presence/absence and to link this to the chronology of spatial occurrence and relative abundance. eDNA collections could facilitate the reconstruction of historical presence and movement of species boundaries (and hybrid zones) with future research avenues including investigating species boundaries, delineating fine‐scale hybrid zones, and tracking the spatiotemporal introgression of invasive genotypes. Importantly, eDNA collections allows for the data and original environmental sample to be stored within long‐term repositories, archived so that new questions may be asked or other taxa within the sample may be studied. The significance of this cannot be understated given the rapid discovery of new markers or genes under selection, rendering eDNA an invaluable tool for evolutionary studies, now and in the future. However, although it is not yet clear whether eDNA analytical methods are best suited for all studies of hybrid zones, applying a combination of approaches will unquestionably provide important insight into species' spatiotemporal population structure and inform downstream analyses of, for example, demography and selection.

## Data Availability

Data sharing is not applicable to this article as no new data were created or analysed in this study.

## References

[mec15514-bib-0001] Adams, C. I. M. , Knapp, M. , Gemmell, N. J. , Jeunen, G.‐J. , Bunce, M. , Lamare, M. D. , & Taylor, H. R. (2019). Beyond biodiversity: Can environmental DNA (eDNA) cut it as a population genetics tool? Genes, 10, 192 10.3390/genes10030192 PMC6470983 30832286

[mec15514-bib-0002] Andersen, K. , Bird, K. L. , Rasmussen, M. , Haile, J. , Breuning‐madsen, H. , Kjaer, K. H. , … Willerslev, E. (2012). Meta‐barcoding of ‘dirt’ DNA from soil reflects vertebrate biodiversity. Molecular Ecology, 21, 1966–1979. 10.1111/j.1365-294X.2011.05261.x 21917035

[mec15514-bib-0003] Aylward, M. L. , Sullivan, A. P. , Perry, G. H. , Johnson, S. E. , & Louis, E. E. (2018). An environmental DNA sampling method for aye‐ayes from their feeding traces. Ecology and Evolution, 2018(8), 9229–9240. 10.1002/ece3.4341 PMC6194247 30377496

[mec15514-bib-0004] Bálint, M. , Pfenninger, M. , Grossart, H.‐P. , Taberlet, P. , Vellend, M. , Leibold, M. A. , … Bowler, D. (2018). Environmental DNA time series in ecology. Trends in Ecology and Evolution, 33(12), 945–957. 10.1016/j.tree2018.09.003 30314916

[mec15514-bib-0005] Barnes, M. A. , & Turner, C. R. (2016). The ecology of environmental DNA and implications for conservation genetics. Conservation Genetics, 17, 1 10.1007/s10592-015-0775-4

[mec15514-bib-0006] Biggs, J. , Ewald, N. , Valentini, A. , Gaboriaud, C. , Dejean, T. , Griffiths, R. A. , … Dunn, F. (2015). Using eDNA to develop a national citizen science‐based monitoring programme for the great crested newt (*Triturus cristatus*). Biological Conservation, 183, 19–28. 10.1016/j.biocon.2014.11.029

[mec15514-bib-0007] Brinkman, T. J. , & Hundertmark, K. J. (2009). Sex identification of northern ungulate using low quality and quantity DNA. Conservation Genetics, 10, 1189 10.1007/s10592-008-9747-2

[mec15514-bib-0008] Buggs, R. (2007). Empirical study of hybrid zone movement. Heredity, 99, 301–312. 10.1038/sj.hdy.6800997 17611495

[mec15514-bib-0009] Buxton, A. , Groombridge, J. , & Griffiths, R. (2018). Comparison of two citizen scientist methods for collecting pond water samples for environmental DNA studies. Citizen Science: Theory and Practice, 3(2), 2.

[mec15514-bib-0010] Bylemans, J. , Furlan, E. M. , Hardy, C. M. , McGuffie, P. , Lintermans, M. , & Gleeson, D. M. (2017). An environmental DNA‐based method for monitoring spawning activity: A case study, using the endangered Macquarie perch (*Macquaria australasica*). Methods in Ecology and Evolution, 8(5), 646–655.

[mec15514-bib-0011] Carpenter, M. L. , Buenrostro, J. D. , Valdiosera, C. , Schroeder, H. , Allentoft, M. E. , Sikora, M. , … Bustamante, C. D. (2013). Pulling out the 1%: Whole‐genome capture for the targeted enrichment of ancient dna sequencing libraries. American Journal of Human Genetics, 93, 852–864. 10.1016/j.ajhg.2013.10.002 24568772 PMC3824117

[mec15514-bib-0012] Curran, J. , Triggs, C. M. , Buckleton, J. , & Weir, B. (1999). Interpreting DNA mixtures in structured populations. Journal of Forensic Sciences, 44, 987–995. 10.1520/JFS12028J 10486951

[mec15514-bib-0013] Deiner, K. , Fronhofer, E. A. , Mächler, E. , Walser, J.‐C. , & Altermatt, F. (2016). Environmental DNA reveals that rivers are conveyer belts of biodiversity information. Nature Communications, 7, 12544 10.1038/ncomms12544 PMC5013555 27572523

[mec15514-bib-0014] Dell'Anno, A. , & Danovaro, R. (2005). Extracellular DNA plays a key role in deep‐sea ecosystem functioning. Science, 309(5744), 2179 10.1126/science.1117475 16195451

[mec15514-bib-0015] Dufresnes, C. , Dejean, T. , Zumbach, S. , Schmidt, B. R. , Fumagalli, L. , Ramseier, P. , & Dubey, S. (2019). Early detection and spatial monitoring of an emerging biological invasion by population genetics and environmental DNA metabarcoding. Conservation Science and Practices, 1, e86 10.1111/csp2.86

[mec15514-bib-0016] Dysthe, J. C. , Franklin, T. W. , McKelvey, K. S. , Young, M. K. , & Schwartz, M. K. (2018). An improved environmental DNA assay for bull trout (*Salvelinus confluentus*) based on the ribosomal internal transcribed spacer 1. PLoS One, 13(11), e0206851. 30399172 10.1371/journal.pone.0206851 PMC6219789

[mec15514-bib-0017] Egan, S. P. , Barnes, M. A. , Hwang, C.‐T. , Mahon, A. R. , Feder, J. L. , Ruggiero, S. T. , … Lodge, D. M. (2013). Rapid invasive species detection by combining environmental DNA with light transmission spectroscopy. Conservation Letters, 6, 402–409. 10.1111/conl.12017

[mec15514-bib-0018] Feder, A. F. , Petrov, D. A. , & Bergland, A. O. (2012). LDx: Estimation of linkage disequilibrium from high‐throughput pooled resequencing data. PLoS One, 7(11), e48588 10.1371/journal.pone.0048588 23152785 PMC3494690

[mec15514-bib-0019] Ficetola, G. F. , Miaud, C. , Pompanon, F. , & Taberlet, P. (2008). Species detection using environmental DNA from water samples. Biology Letters, 4(4), 423–425. 10.1098/rsbl.2008.0118 18400683 PMC2610135

[mec15514-bib-0020] Ficetola, G. F. , Poulenard, J. , Sabatier, P. , Messager, E. , Gielly, L. , Leloup, A. , … Arnaud, F. (2018). DNA from lake sediments reveals long‐term ecosystem changes after a biological invasion. Science Advances, 4(5), 9 10.1126/sciadv.aar4292 PMC5942909 29750197

[mec15514-bib-0021] Foote, A. D. , Thomsen, P. F. , Sveegaard, S. , Wahlberg, M. , Kielgast, J. , Kyhn, L. A. , … Gilbert, M. T. P. (2012). Investigating the potential use of environmental DNA (eDNA) for genetic monitoring of marine mammals. PLoS One, 7(8), e41781 10.1371/journal.pone.0041781 22952587 PMC3430683

[mec15514-bib-0022] Franklin, T. W. , McKelvey, K. S. , Golding, J. D. , Mason, D. H. , Dysthe, J. C. , Pilgrim, K. L. , … Schwartz, M. K. (2019). Using environmental DNA methods to improve winter surveys for rare carnivores: DNA from snow and improved noninvasive techniques. Biological Conservation, 229, 50–58. 10.1016/j.biocon.2018.11.006

[mec15514-bib-0023] Goldberg, C. S. , Turner, C. R. , Deiner, K. , Klymus, K. E. , Thomsen, P. F. , Murphy, M. A. , … Taberlet, P. (2016). Critical considerations for the application of environmental DNA methods to detect aquatic species. Methods Ecology and Evolution, 7, 1299–1307.

[mec15514-bib-0024] Gompert, Z. , Mandeville, L. , & Buerkle, C. A. (2017). Analysis of population genomic data from hybrid zones. Annual Review of Ecology, Evolution, and Systematics, 48, 207–229. 10.1146/annurev-ecolsys-110316-022652

[mec15514-bib-0025] Gorički, Š. , Stanković, D. , Snoj, A. , Kuntner, M. , Jeffery, W. R. , Trontelj, P. , … Aljančič, G. (2017). Environmental DNA in subterranean biology: Range extension and taxonomic implications for Proteus. Scientific Reports, 7, 45054 10.1038/srep45054 28345609 PMC5366867

[mec15514-bib-0026] Grabenstein, K. , & Taylor, S. A. (2018). Breaking barriers: Causes, consequences, and experimental utility of human‐mediated hybridization. Trends in Ecology and Evolution, 33, 198–212. 10.1016/j.tree.2017.12.008 29306562

[mec15514-bib-0027] Haned, H. , Pène, L. , Lobry, J. R. , Dufour, A. B. , & Pontier, D. (2011). Estimating the number of contributors to forensic DNA mixtures: Does maximum likelihood perform better than maximum allele count? Journal of Forensic Sciences, 56, 23–28. 10.1111/j.1556-4029.2010.01550.x 20840286

[mec15514-bib-0028] Harrison, R. G. (1990). Hybrid zones: Windows on evolutionary process. Oxford Surveys in Evolutionary Biology, 7, 69–128.

[mec15514-bib-0029] Harrison, R. G. , & Larson, E. L. (2014). Hybridization, introgression, and the nature of species boundaries. Journal of Heredity, 105, 795–809. 10.1093/jhered/esu033 25149255

[mec15514-bib-0030] Hohenlohe, P. A. , Day, M. D. , Amish, S. J. , Miller, M. R. , Kamps‐Hughes, N. , Boyer, M. C. , … Luikart, G. (2013). Genomic patterns of introgression in rainbow and westslope cutthroat trout illuminated by overlapping paired‐end RAD sequencing. Molecular Ecology, 22, 3002–3013. 10.1111/mec.12239 23432212 PMC3664261

[mec15514-bib-0031] Holman, L. E. , Hollenbeck, C. M. , Ashton, T. J. , & Johnston, I. A. (2019). Demonstration of the use of environmental DNA for the non‐invasive genotyping of a bivalve Mollusk, the European Flat Oyster (*Ostrea edulis*). Frontiers in Genetics, 10, 1159 10.3389/fgene.2019.01159 31803238 PMC6877716

[mec15514-bib-0032] Jerde, C. L. , Mahon, A. R. , Chadderton, W. L. , & Lodge, D. M. (2011). “Sight‐unseen” detection of rare aquatic species using environmental DNA. Conservation Letters, 4, 150–157. 10.1111/j.1755-263X.2010.00158.x

[mec15514-bib-0033] Kelly, R. P. , Port, J. A. , Yamahara, K. M. , & Crowder, L. B. (2014). Using environmental DNA to census marine fishes in a large mesocosm. PLoS One, 9, e86175 10.1371/journal.pone.0086175 24454960 PMC3893283

[mec15514-bib-0034] Kirkpatrick, J. B. , Walsh, E. A. , & D'Hondt, S. (2016). Fossil DNA persistence and decay in marine sediment over hundred‐thousand‐year to million‐year time scales. Geology, 44(8), 615–618. 10.1130/G37933.1

[mec15514-bib-0035] Laramie, M. B. , Pilliod, D. S. , & Goldberg, C. S. (2015). Characterizing the distribution of an endangered salmonid using environmental DNA analysis. Biological Conservation, 183, 29–37. 10.1016/j.biocon.2014.11.025

[mec15514-bib-0036] Larson, E. L. , Andres, J. A. , Bogdanowicz, S. B. , & Harrison, F. (2013). Differential introgression in a mosaic hybrid zone reveals candidate barrier genes. Evolution, 67, 3653–3661. 10.1111/evo.12205 24299416

[mec15514-bib-0037] Levin, D. A. (2006). The spatial sorting of ecological species: Ghost of competition or of hybridization past? Systematic Botany, 31, 8–12. 10.1600/036364406775971831

[mec15514-bib-0038] Ma, H. , Stewart, K. A. , Lougheed, S. C. , Zheng, J. , Wang, Y. , & Zhao, J. (2016). Characterization, optimization, and validation of environmental DNA (eDNA) markers to detect endangered aquatic mammals. Conservation Genetic Resources, 8(4), 561–568. 10.1007/s12686-016-0597-9

[mec15514-bib-0039] Mallet, J. , Besansky, N. , & Hahn, M. W. (2016). How reticulated are species? BioEssays, 38, 140–149. 10.1002/bies.201500149 26709836 PMC4813508

[mec15514-bib-0040] Mandeville, E. G. , Parchman, T. L. , Thompson, K. G. , Compton, R. I. , Gelwicks, K. R. , Song, S. J. , & Buerkle, C. A. (2017). Inconsistent reproductive isolation revealed by interactions between Catostomus fish species. Evolutionary Letters, 1, 255–268. 10.1002/evl3.29 PMC6121845 30283654

[mec15514-bib-0041] McEntee, J. P. , Burleigh, J. G. , & Singhal, S. (2018). Dispersal predicts hybrid zone width across animal diversity: Implications for species borders under incomplete reproductive isolation. bioRxiv. 10.1101/472506 32552108

[mec15514-bib-0042] Minamoto, T. , Uchii, K. , Takahara, T. , Kitayoshi, T. , Tsuji, S. , Yamanaka, H. , & Doi, H. (2017). Nuclear internal transcribed spacer‐1 as a sensitive genetic marker for environmental DNA studies in common carp *Cyprinus carpio* . Molecular Ecology Resources, 17, 324–333. 27487846 10.1111/1755-0998.12586

[mec15514-bib-0043] Mioduchowska, M. , Kaczmarczyk, A. , Zając, K. , Zając, T. , & Sell, J. (2016). Gender‐associated mitochondrial DNA heteroplasmy in somatic tissues of the endangered freshwater mussel Unio crassus (Bivalvia: Unionidae): Implications for sex identification and phylogeographic studies. Journal of Experimental Zoology Part A: Ecological Genetics and Physiology, 325A, 610–625. 10.1002/jez.2055 28102008

[mec15514-bib-0044] Muha, T. P. , Rodríguez‐Rey, M. , Rolla, M. , & Tricarico, E. (2017). Using environmental DNA to improve species distribution models for freshwater invaders. Frontiers in Ecology and Evolution, 5, 158 10.3389/fevo.2017.00158

[mec15514-bib-0045] Nelson‐Chorney, H. T. , Davis, M. S. , Poesch, M. S. , Vinebrooke, R. D. , Carli, C. M. , & Taylor, M. K. (2019). Environmental DNA in lake sediment reveals biogeography of native genetic diversity. Frontiers in Ecology and the Environment, 17(6), 313–318. 10.1002/fee.2073

[mec15514-bib-0046] Payseur, B. A. , & Rieseberg, L. H. (2016). A genomic perspective on hybridization and speciation. Molecular Ecology, 25(11), 2337–2360. 26836441 10.1111/mec.13557 PMC4915564

[mec15514-bib-0047] Pfenninger, M. , Patel, S. , Arias‐Rodriguez, L. , Feldmeyer, B. , Riesch, R. , & Plath, M. (2015). Unique evolutionary trajectories in repeated adaptation to hydrogen sulphide‐toxic habitats of a neotropical fish (*Poecilia mexicana*). Molecular Ecology, 24(21), 5446–5459. 26405850 10.1111/mec.13397

[mec15514-bib-0048] Pietramellara, G. , Ascher, J. , Borgogni, F. , Ceccherini, M. T. , Guerri, G. , & Nannipieri, P. (2009). Extracellular DNA in soil and sediment: Fate and ecological relevance. Biology and Fertility of Soils, 45(3), 219–235. 10.1007/s00374-008-0345-8

[mec15514-bib-0049] Pilliod, D. S. , Goldberg, C. S. , Arkle, R. S. , & Waits, L. P. (2014). Factors influencing detection of eDNA from a stream‐dwelling amphibian. Molecular Ecology Resources, 14, 109–116. 10.1111/1755-0998.12159 24034561

[mec15514-bib-0050] Pinfield, R. , Dilane, E. , Runge, A. K. W. , Evans, A. , Mirimin, L. , Niemann, J. , … Foote, A. D. (2019). False‐negative detections from environmental DNA collected in the presence of large numbers of killer whales (*Orcinus orca*). Environmental DNA, 1, 316–328.

[mec15514-bib-0051] Pochon, X. , Zaiko, A. , Fletcher, L. M. , Laroche, O. , & Wood, S. A. (2017). Wanted dead or alive? Using metabarcoding of environmental DNA and RNA to distinguish living assemblages for biosecurity applications. PLoS One, 12(11), e0187636 10.1371/journal.pone.0187636 29095959 PMC5667844

[mec15514-bib-0052] Qu, C. , & Stewart, K. A. (2019). Evaluating monitoring options for conservation: Traditional and environmental DNA tools for a critically endangered mammal. Science of Nature: Naturwissenschaften, 106, 9 10.1007/s00114-019-1605-1 30778682

[mec15514-bib-0053] Rafati, N. , Blanco‐Aguiar, J. A. , Rubin, C. J. , Sayyab, S. , Sabatino, S. J. , Afonso, S. , … Carneiro, M. (2018). A genomic map of clinal variation across the European rabbit hybrid zone. Molecular Ecology, 27, 1457–1478. 10.1111/mec.14494 29359877

[mec15514-bib-0054] Ryan, S. F. , Deines, J. M. , Scriber, J. M. , Pfrender, M. E. , Jones, S. E. , Emrich, S. J. , & Hellmann, J. L. (2018). Climate‐mediated hybrid zone movement revealed with genomics, museum collection, and simulation modeling. Proceedings of the National Academy of Sciences of the United States of America, 115(10), E2284–E2291. 10.1073/pnas.1714950115 29463695 PMC5877999

[mec15514-bib-0055] Schumer, M. , Powell, D. L. , Declos, P. J. , Squire, M. , Cui, R. , Andolfatto, P. , & Rosenthal, G. G. (2017). Assortative mating and persistent reproductive isolation in hybrids. Proceedings of the National Academy of Sciences of the United States of America, 114, 10936–10941. 10.1073/pnas.1711238114 28973863 PMC5642718

[mec15514-bib-0056] Scriber, J. M. (2011). Impacts of climate warming on hybrid zone movement: Geographically diffuse and biologically porous “species borders”. Insect Science, 18, 121–159. 10.1111/j.1744-7917.2010.01367.x

[mec15514-bib-0057] Sethi, S. A. , Larson, W. , Turnquist, K. , & Isermann, D. (2018). Estimating the number of contributors to DNA mixtures provides a novel tool for ecology. Methods in Ecology and Evolution, 10, 109–119.

[mec15514-bib-0058] Sigsgaard, E. E. , Jensen, M. R. , Winkelmann, I. E. , Møller, P. R. , Hansen, M. M. , & Thomsen, P. F. (2020). Population‐level inferences from environmental DNA—Current status and future perspectives. Evolutionary Applications, 13, 245–262. 10.1111/eva.12882 31993074 PMC6976968

[mec15514-bib-0059] Sigsgaard, E. E. , Nielsen, I. B. , Bach, S. S. , Lorenzen, E. D. , Robinson, D. P. , Knudsen, S. W. , … Thomsen, P. F. (2017). Population characteristics of a large whale shark aggregation inferred from seawater environmental DNA. Nature Ecology and Evolution, 1, 4 10.1038/s41559-016-0004 28812572

[mec15514-bib-0060] Spear, S. F. , Groves, J. D. , Williams, L. A. , & Waits, L. P. (2015). Using environmental DNA methods to improve detectability in a hellbender (*Cryptobranchus alleganiensis*) monitoring program. Biological Conservation, 183, 38–45. 10.1016/j.biocon.2014.11.016

[mec15514-bib-0061] Steven, B. , Hesse, C. , Soghigian, J. , Gallegos‐Graves, V. , & Dunbar, J. (2017). Simulated rRNA/DNA ratios show potential to misclassify active populations as dormant. Applied Environmental Microbiology, 83(11), e00696‐17 10.1128/AEM.00696-17 28363969 PMC5440720

[mec15514-bib-0062] Stewart, K. A. (2019). Understanding the effects of biotic and abiotic factors on sources of aquatic environmental DNA. Biodiversity and Conservation, 28(5), 983–1001. 10.1007/s10531-019-01709-8

[mec15514-bib-0063] Stewart, K. A. , Austin, J. D. , Zamudio, K. R. , & Lougheed, S. C. (2016). Contact zone dynamics during the early stages of speciation in a chorus frog (*Pseudacris crucifer*). Heredity, 116(2), 239–247. 26626576 10.1038/hdy.2015.96 PMC4806893

[mec15514-bib-0064] Stewart, K. A. , Hudson, C. , & Lougheed, S. C. (2017). Can alternative male mating tactics facilitate introgression across a hybrid zone by circumventing female choice? Journal of Evolutionary Biology, 30(2), 412–421. 27862550 10.1111/jeb.13017

[mec15514-bib-0065] Stewart, K. A. , Ma, H. , Zheng, J. , & Zhao, J. (2017). Using environmental DNA to assess population‐wide spatiotemporal reserve use. Conservation Biology, 31(5), 1173–1182. 10.1111/cobi.12910 28221696

[mec15514-bib-0066] Taberlet, P. , Mattock, H. , Dubois‐Paganon, C. , & Bouvet, J. (1993). Sexing free‐ranging brown bears *Ursus arctos* using hairs found in the field. Molecular Ecology, 2(6), 399–403. 10.1111/j.1365-294X.1993.tb00033.x 8162229

[mec15514-bib-0067] Taus, T. , Futschik, A. , & Schlötterer, C. (2017). Quantifying selection with pool‐seq time series data. Molecular Biology and Evolution, 34(11), 3023–3034. 10.1093/molbev/msx225 28961717 PMC5850601

[mec15514-bib-0068] Taylor, S. A. , & Larson, E. L. (2019). Insights from genomes into the evolutionary importance and prevalence of hybridization in nature. Nature Ecology and Evolution, 3, 170–177. 10.1038/s41559-018-0777-y 30697003

[mec15514-bib-0069] Taylor, S. A. , Larson, E. L. , & Harrison, R. G. (2015). Hybrid zones: Windows on climate change. Trends in Ecology and Evolution, 30, 398–406. 10.1016/j.tree.2015.04.010 25982153 PMC4794265

[mec15514-bib-0070] Thomsen, P. F. , Kielgast, J. , Iversen, L. L. , Moller, P. R. , Rasmussen, M. , & Willerslev, E. (2012). Detection of a diverse marine fish fauna using environmental DNA from seawater samples. PLoS One, 7, e41732 10.1371/journal.pone.0041732 22952584 PMC3430657

[mec15514-bib-0071] Uchii, K. , Doi, H. , & Minamoto, T. (2016). A novel environmental DNA approach to quantify the cryptic invasion of non‐native genotypes. Molecular Ecology Resources, 16, 415–422. 10.1111/1755-0998.12460 26307935

[mec15514-bib-0072] Uchii, K. , Doi, H. , Yamanaka, H. , & Minamoto, T. (2017). Distinct seasonal migration patterns of Japanese native and non‐native genotypes of common carp estimated by environmental DNA. Ecology and Evolution, 7(20), 8515–8522. 10.1002/ece3.3346 29075467 PMC5648683

[mec15514-bib-0073] Ushio, M. , Fukuda, H. , Inoue, T. , Makoto, K. , Kishida, O. , Sato, K. , … Miya, M. (2017). Environmental DNA enables detection of terrestrial mammals from forest pond water. Molecular Ecology Resources, 17(6), 63–75. 10.1111/1755-0998.12690 28603873

[mec15514-bib-0074] Weir, B. S. , Triggs, C. M. , Starling, L. , Stowell, L. I. , Walsh, K. A. J. , & Buckleton, J. (1997). Interpreting DNA mixtures. Journal of Forensic Sciences, 42, 213–222. 10.1520/JFS14100J 9068179

[mec15514-bib-0075] Wilcox, T. M. , Young, M. K. , McKelvey, K. S. , Isaak, D. J. , Horan, D. L. , & Schwartz, M. K. (2018). Fine‐scale environmental DNA sampling reveals climate‐mediated interactions between native and invasive trout species. Ecosphere, 9(11), e02500 10.1002/ecs2.2500

[mec15514-bib-0076] Willerslev, E. , Cappellini, E. , Boomsma, W. , Nielsen, R. , Hebsgaard, M. B. , Brand, T. B. , … Collins, M. J. (2007). Ancient biomolecules from deep ice cores reveal a forested southern Greenland. Science, 317(5834), 111–114. 17615355 10.1126/science.1141758 PMC2694912

[mec15514-bib-0077] Williams, M.‐A. , O'Grady, J. , Ball, B. , Carlsson, J. , Eyto, E. , McGinnity, P. , … Parle‐McDermott, A. (2019). The application of CRISPR‐Cas for single species identification from environmental DNA. Molecular Ecology Resources, 19(5), 1106–1114. 10.1111/1755-0998.13045 31177615

